# Supplementary material for: Picometer polar atomic displacements in strontium titanate determined by resonant X-ray diffraction
Source: Nat Commun. 2018 Jan 12;9:178. doi: 10.1038/s41467-017-02599-6 (PMC5766522; doi:10.1038/s41467-017-02599-6)
Supplement: Supplementary file 1 — Supplementary Information [file 41467_2017_2599_MOESM1_ESM.pdf]

Supplementary information

Supplementary Note 1: X-ray absorption fine structure

Since we propose a method for crystal structure determination that relies on resonant X-ray diffraction (RXD) measurements, it should be considered that the fine structure in $f'(E)$ and $f''(E)$ (see Eq. (4) of the main text) also depends on the crystal structure at energies close to the absorption edge. The safest way to obtain the correct fine structure for modeling RXD is to measure the absorption spectra (f'') and use Kramers-Kronig transform to get the dispersion correction in f'. In this work, we deduced the dispersion fine structure for cubic $SrTiO_3$ and assumed it remains unchanged during the phase transition to the tetragonal MFP phase. This was based on two observations. Firstly, the fine structure has a relatively weak influence on the energy dependencies of the Bragg reflections that were the basis for our analysis. This is attributable to the fact that the very strong features in the data were, as expected, found in the pre-edge region – at energies below the edge where fine structure does not play a role. Secondly, we performed X-ray absorption near edge spectroscopy (XANES) measurements at the titanium K-edge to study the structure of the MFP phase beforehand. As it is located in the near surface region of the sample, the MFP phase can be selected by variation of the incidence angle of the X-rays and, hence, adjusting their penetration depth. We then obtained the absorption spectra in fluorescence mode. The resulting XANES spectra only show faint changes upon formation of the MFP phase (see Supplementary Figure 1). At

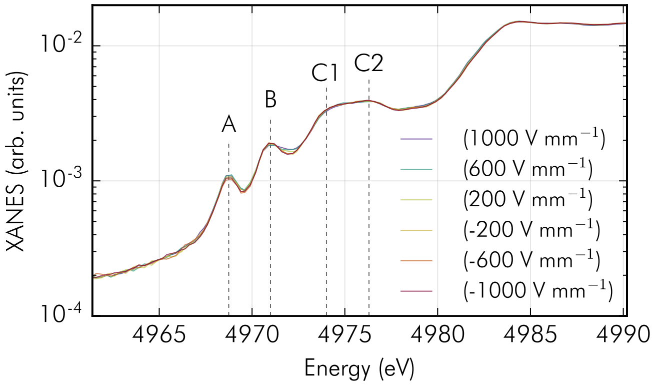

Supplementary Figure 1: Development of the Ti-K edge XANES during formation of the MFP phase
The formation was controlled by stepwise increase of the electric field from $-1000 \ldots 1000\,\mathrm{V\,mm^{-1}}$. Marked are the typical pre-edge features[1;2] known for $SrTiO_3$. A slight increase of absorption between features B and C1 is apparent as well as a decrease of peak A.

the Sr-K edge, on the other hand, no changes were observed since no pre-edge features are expressed which, in contrast to the Ti d-states, is due to a lack of local density of states at the conduction band minimum. The very small effect of formation of the MFP phase on the Ti-K XANES can be explained by the small atomic displacements that are involved. XANES is typically used to study displacements that are more than 10 times larger[3;2].

Supplementary Note 2: Range of application

From the methodological point of view, the question arises whether the method based on resonant suppression of X-ray diffraction can be applied to other crystal structures, in particular, which prerequisites it entails and how likely it is to find suitable reflections. The amount of available reflections grows with higher photon energies, larger unit cells and lower symmetry. On the other

hand, high energies require heavy atoms to allow resonant measurements and too large unit cells lead to a large number of independent atoms which could render a full refinement of all parameters exceedingly difficult.

Nevertheless, there is a large set of structures remaining that fits the conditions for an application of the method, among which each material needs to be assessed individually. To give an outlook on the probability to find suitable reflections and perform similar measurements, we analyzed a large random set of structures taken from the Crystallography Open Database[4] (COD) having the following characteristics: a unit cell smaller than 2000 $\mathrm{\AA}^3$, a limited number of four different elements and at least one element heavier than potassium. For this set we compared calculated RXD spectra in the $5\ldots100\,\mathrm{keV}$ range of all reflections having a momentum transfer of up to $2\sin\theta/\lambda \leq 3\,\mathrm{\AA}^{-1}$ before and after imposing an isotropic mean square displacement of $U_{\mathrm{iso}} = 0.0001\,\mathrm{\AA}^2$ onto the heaviest atom. Among other parameters, we looked at the reflection yielding the maximum contrast $(\Delta I/I)_{\mathrm{max}}$ in intensity over the whole energy range and compared it to the present case of $SrTiO_3$. The resulting distribution is shown in Supplementary Figure 2. The obtained values extend over a

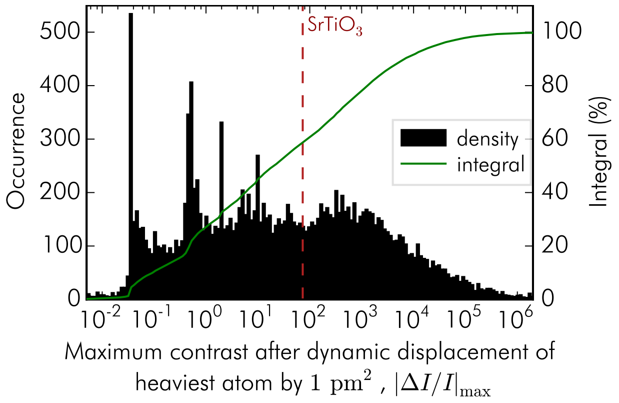

Supplementary Figure 2: Contrast of the method when applied to structures in comparison to $SrTiO_3$ Distribution of maximum observed relative intensity changes calculated for a random set of structures after introducing a mean square displacement on one atom. The dashed line marks the present case of $SrTiO_3$.

wide range. It is clear that the method cannot be applied routinely to all structures fulfilling the conditions mentioned above. However, $SrTiO_3$ is only an average representative of the described set – almost half of the processed samples have reflections that show a larger response to the dynamic displacement. This observation is very promising for future studies that could answer open questions in structure analysis or reveal structure dynamics.

It should be highlighted that the presented development directly complements existing methods of crystal structure determination. Spectroscopic techniques, such as diffraction anomalous fine structure (DAFS) or X-ray absorption fine structure (XAFS), do not discriminate whether distance changes are a result of lattice strain or molecule deformation. Moreover, XAFS does not even allow for lattice parameter selective probing. These techniques can therefore not provide the sub-picometer precision for atomic displacement we aimed for. The routine methods of X-ray crystallography, based on pure Bragg diffraction, including the multi-wavelength anomalous dispersion (MAD) only reach picometer resolution of atomic positions by measuring a very high number of isolated Bragg reflections and require very homogeneous samples.

The analysis of crystal truncation rods (CTRs) in reciprocal space bears similarity to the presented method, since in both cases the substantial variations in the signal are observed at the slopes, away from intensity maxima. In the body centered unit cell, a selection rule exists for the set of Bragg reflections that was in focus for our analysis (one odd miller index). This means they

would be precisely zero if the two metal cations (Sr, Ti) would have the same scattering amplitude. This can also be referred to as anti-Bragg condition where surface contributions (CTR) to the scattered intensity become important. When we use the energy dependence to reduce the Sr scattering amplitude, we come close to this situation and, in principle, CTR contributions may play a role. However, the fundamental difference is that we maintain the Bragg condition for each selected crystalline region in our case of resonantly suppressed diffraction (RSD): The enormous sensitivity to positional changes of atoms is achieved through the analysis of energy dependencies near destructive interference effects while we reside at a fixed position of reciprocal space. Therefore, the CTR would only result in a constant offset of the whole spectrum. In our case it was it was not necessary to take such contribution into account, because even though the Bragg intensity at the minimum is reduced by several orders of magnitude, it is still much stronger than the surface scattering. This assessment can be made based on the clear appearance of a Bragg maximum from cubic $SrTiO_3$ even at the energy of destructive interference (see black crosses in Fig. 6 of the main text).

Since it relies on thin layers having a well defined truncation (interface), the CTR method would not be suitable for structure determination of the MFP phase which is likely to be distributed inhomogeneously as the formation takes place along vacancy migration paths. With our approach, on the other hand, we can determine the structure of layers that are potentially buried, selecting each individual layer according to its lattice constants. The probed regions may be inhomogeneous and knowledge of the morphology is not necessary.

Finally, methods like Grazing incidence diffraction (GID) or reciprocal space mapping (RSM) utilize interference effects to investigate thickness and strain in layered or 3D structured systems, but are usually not sensitive enough to detect such small atomic displacements. In fact, a combination of these techniques with the RSD approach can be a powerful tool for investigation of complex objects.

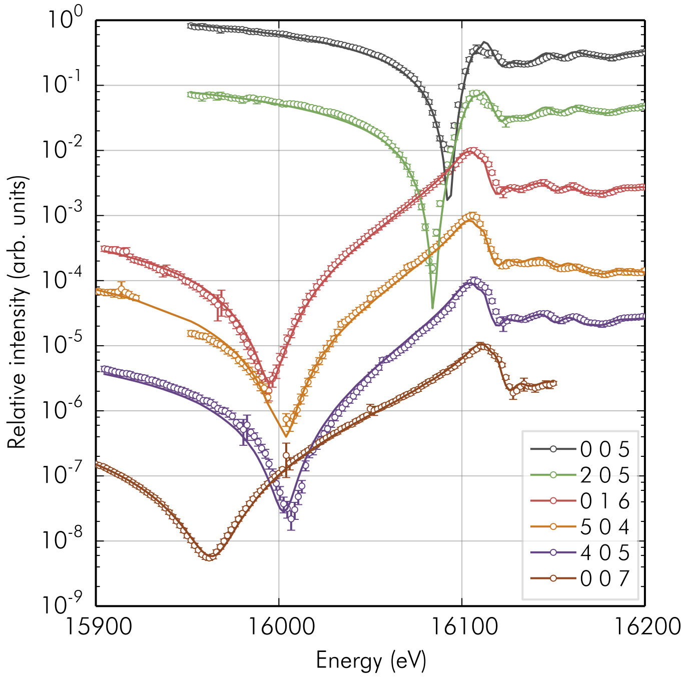

Supplementary Figure 3: Energy dependence for several Bragg reflections of bulk $SrTiO_3$
These data have been acquired under normal conditions and with no electric field applied to characterize the initial state of the single crystals. Open circles are measured values and solid lines show the calculation after refinement of Debye-Waller factors. The curves have successively been scaled by a factor of 10 in the same order as they appear in the legend. Error bars indicate the standard deviation calculated from the incident and diffracted beam intensity.

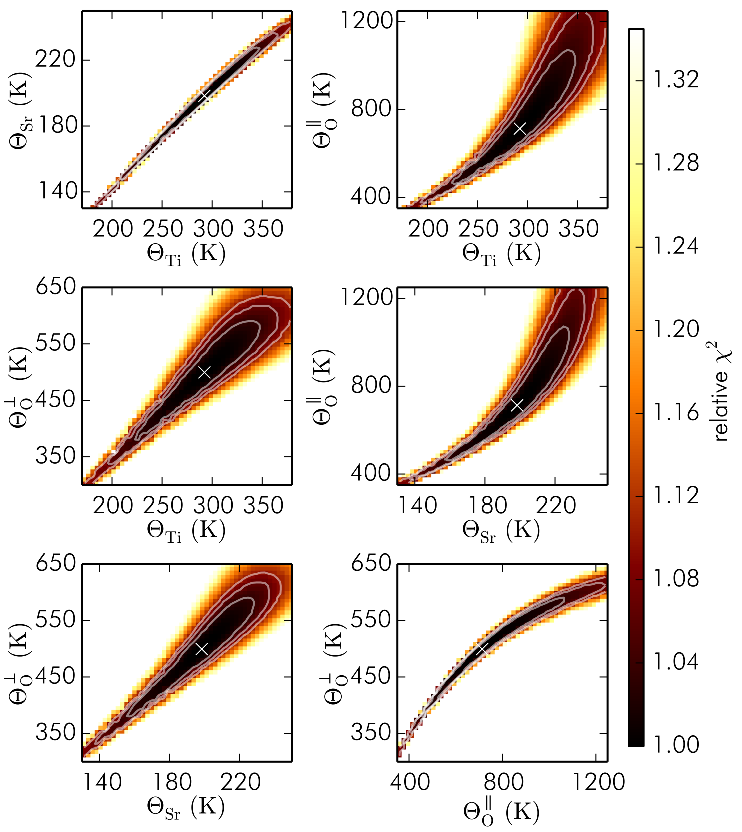

Supplementary Figure 4: Confidence intervals of the fitted Debye temperatures
Correlations are illustrated by the dependence of the residual sum of squares χ^2 on pairwise variation of the fit parameters (Debye temperaturesk). White crosses indicate the global minima. Confidence regions for 1σ, 2σ and 3σ are marked by white contour lines.

Supplementary References

[1] Vedrinskii, R. V., Kraizman, V. L., Novakovich, A. A., Demekhin, P. V. & Urazhdin, S. V. Pre-edge fine structure of the 3d atom k x-ray absorption spectra and quantitative atomic structure determinations for ferroelectric perovskite structure crystals. *Journal of Physics: Condensed Matter* **10**, 9561–9580 (1998). URL `http://dx.doi.org/10.1088/0953-8984/10/42/021`.

[2] Stöcker, H. *et al.* Surface-near modifications of SrTiO3 local symmetry due to nitrogen implantation investigated by grazing incidence XANES. *Scripta Materialia* **86**, 1–4 (2014). URL `https://doi.org/10.1016/j.scriptamat.2014.02.014`.

[3] Frenkel, A. I. *et al.* Origin of polarity in amorphous srtio$_3$. *Phys. Rev. Lett.* **99**, 215502 (2007). URL `https://link.aps.org/doi/10.1103/PhysRevLett.99.215502`.

[4] Grazulis, S. *et al.* Crystallography open database (COD): an open-access collection of crystal structures and platform for world-wide collaboration. *Nucleic Acids Research* **40**, D420–D427 (2011). URL `http://dx.doi.org/10.1093/nar/gkr900`.
